# Impact of improper sample handling on Cobas CT/NG testing platform with dual swab sample collection kit for detecting *Chlamydia trachomatis* and *Neisseria gonorrhoeae*

**DOI:** 10.1128/spectrum.03224-23

**Published:** 2023-12-04

**Authors:** Dhammika H. Navarathna, Kelle B. Sayers, Derek C. Murray

**Affiliations:** 1 Department of Pathology, Laboratory Medicine Services, Central Texas Veterans Health Care System, Temple, Texas, USA; University of California, San Diego, La Jolla, California, USA

**Keywords:** Cobas, CT/NG, duel swab, invalid

## Abstract

In the realm of molecular diagnostics, accurate and reliable test results are imperative for effective clinical decision-making. This study delves into the effects of non-compliant sample collection practices on the Cobas CT/NG testing platform. Deviating from the manufacturer’s recommendation of single swab collection, our laboratory received dual swabs during sample collection. Subsequently, we embarked on an investigation to ascertain the cause behind a noticeable rise in invalid results, which were found to be exacerbated by improper sample handling practices, accounting for a significant 30% of cases. Through a comprehensive comparative analysis, we found that the use of dual swabs in testing contributed to a substantial increase in both invalid and false-negative results when compared to properly collected samples submitted with a single swab. This study underscores the importance of adhering to recommended protocols for sample collection to ensure accurate and reliable molecular diagnostic outcomes.

**IMPORTANCE:**

The importance of this observation lies in its potential to directly impact testing outcomes and patient care. By identifying improper sample handling as a contributing factor to a substantial number of invalid results, we emphasize the need for meticulous adherence to recommended protocols during sample collection. Laboratories that overlook or are unaware of such deviations may inadvertently compromise the reliability and efficacy of their diagnostic processes, leading to misdiagnoses, delayed treatment, and patient dissatisfaction.

## OBSERVATION

The Cobas CT/NG test is a molecular diagnostic test used to detect two common sexually transmitted infections: *Chlamydia trachomatis* (CT) and *Neisseria gonorrhoeae* (NG) (1). This highly sensitive and specific test utilizes nucleic acid amplification technology to identify the presence of genetic material from these bacteria in patient samples. The Cobas CT/NG test is a valuable tool for early detection and accurate diagnosis of these infections, aiding healthcare providers in providing timely treatment and preventing further transmission (2).

The Cobas CT/NG test employs a dual swab sample kit within its Cobas PCR system, designed for the detection of CT and NG (3). It is worth noting that proper sample collection is crucial for accurate results (4). The collection process involves utilizing a flocked swab specifically for endocervical samples, while woven swabs are employed for collecting other specimens. Cobas CT/NG is validated for use with male and female urine, clinician-instructed self-collected vaginal swab specimens, clinician-collected vaginal swab specimens, anorectal swab specimens, oropharyngeal swab specimens, and endocervical swab specimens, all collected in Cobas PCR Media (Roche Molecular Systems, Inc.). Only one such collected swab is supposed to be in the collection tube (package insert). Adequate understanding of the package instructions, especially regarding the proper use of the swabs, is essential to ensure successful and accurate sample collection, minimizing complications and enhancing the reliability of the test results (3).

Despite repeated efforts to provide educational guidance through memorandums and PowerPoint presentations, our lab continues to receive improperly collected samples by adding both types of swabs inside the collection tube. We observed that improperly collected samples lead to lots of invalid results. This issue might benefit from further investigation into the specific challenges faced by the reporting lab. Hence, in response to this ongoing challenge, a thorough investigation was conducted using 5 months of retrospective data. It was discovered that improperly collected samples, where both types of swabs were placed within the PCR media tube, were leading to the generation of invalid results.

We employed a retrospective approach, utilizing 5-month worth of patient testing data for CT and NG. The data encompassed the total number of tests conducted, including positives, negatives, and invalids. Our analysis included segregating the results based on whether the samples were correctly or incorrectly collected, as well as instances leading to invalid results.

For data analysis, we employed the chi-square test using GraphPad Prism version 10.0.0 for Windows. We assumed that each test was independent, and we analyzed counts from unpaired subjects. This statistical approach allowed us to assess the significance of associations and differences among the various categories within our collected data as shown in Table 1.

**TABLE 1:** Three outcomes due to properly and improperly collected samples and statistics data associated with contingency table

	Correct collection	Incorrect collection
Positives	1,906	791
Negatives	92	24
Invalids	12	123
*P* value and statistical significance	Data analysis	
Test	Chi-square	
Chi-square, df	233.1, 2	
*P* value	<0.0001	
*P-*value summary	****	
One- or two-sided	NA	
Statistically significant (*P* < 0.05)?	Yes	
Data analyzed		
Number of rows	3	
Number of columns	2	

**Fig 1:**
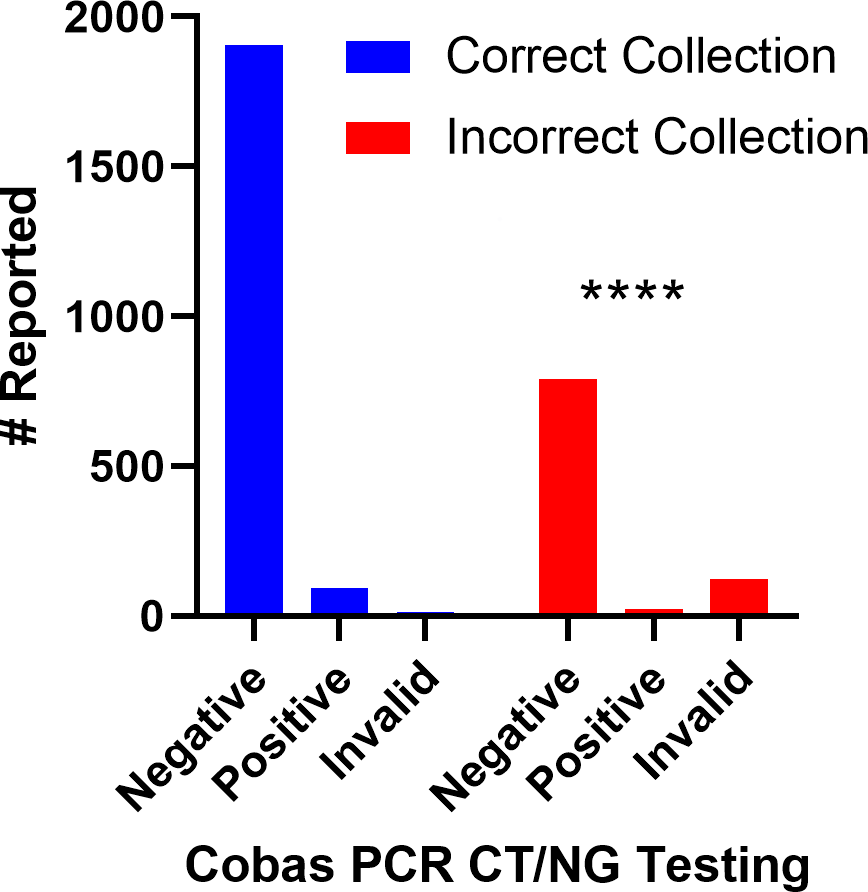
Cobas CT/NG testing conducted over a 5-month duration. The blue bars represent single swabs containing collection tubes, while the red bars represent instances where two swabs were incorrectly added to the sample tubes.

A total of 2,948 specimens for CT/NG testing were collected over a span of 5 months, from March 2023 to July 2023. Among these, 938 samples (31%) were collected incorrectly having dual swabs, falling outside the manufacturers’ instructions. This is associated with a substantial number of invalid results, with 123 specimens (13.1%) showing invalid outcomes due to improper collection. In contrast, only 12 invalid results (0.6%) were observed in 2,010 samples that were correctly collected (Fig. 1). The analysis also revealed that 92 positive samples (4.5%) were detected in properly collected specimens, while 24 samples (2.5%) yielded positive results from improperly collected specimens (Fig. 1).

The chi-square test and Fisher’s exact test showed a highly significant association between proper collection techniques and accurate outcomes. The calculated *P* value was found to be less than 0.0001, indicating a robust statistical significance. This suggests that properly collected samples are statistically associated with accurate results compared to improperly collected samples in the Cobas PCR dual swab sample kit.

Our observation and follow-up study not only validate our hypothesis of increased variability in test results due to the use of two swabs in the collection tubes but also indicate compromised precision and accuracy of the test (Fig. 1), posing a risk to patient testing. Addressing this lack of clarity or complexity would involve considering the exclusion of such samples in future testing. A subsequent study will further validate our findings in this regard. This emphasized the critical importance of following proper collection procedures to ensure the accuracy of test outcomes. Effective communication and continuous training remain essential to rectify this issue and maintain the integrity of the Cobas CT/NG test results. Additional training, hands-on demonstrations, or more visual aids could potentially help address the problem and improve the overall sample collection process.

The lack of establishing sensitivity and specificity together with precision calculations using dual swabs in positive and negative samples is a limitation of this study. However, it is beyond the scope of this observation as we assume to notify our peer clinical community.

Broad clinical performances, validation, and verifications of the Cobas CT/NG assay on the Cobas 6800/8800 systems (Cobas) for the detection of CT and NG were previously published in a multisite, prospective study using male and female urogenital specimens. This test is considered one of the cutting-edge high throughputs and a highly sensitive and precise test (2).

Overall, the results underscore the importance of adhering to manufacturers’ instructions for sample collection, as improper collection techniques were associated with a significantly higher rate of invalid results and false-negative outcomes (Fig. 1). These findings emphasize the critical role of proper collection practices in ensuring the reliability of CT/NG test results.

Due to a disproportionally high number of invalid test results associated with samples received with two swabs in a single collection tube, lab will begin rejecting CT/NG samples containing two swabs in a single tube beginning 1 October 2023 per manufacturer recommendation.

Our data mining approach allowed us to examine the impact of proper sample collection on test outcomes and evaluate the significance of accurate collection techniques in obtaining reliable and valid results in Cobas PCR dual swab sample kit for CT and NG. The utilization of the chi-square test in conjunction with GraphPad Prism enabled us to draw meaningful conclusions from our data set and gain insights into the relationship between sample collection methods and the observed outcomes.
